# Supplementary material for: The influence of visitor-based social contextual information on visitors’ museum experience
Source: PLoS One. 2022 May 24;17(5):e0266856. doi: 10.1371/journal.pone.0266856 (PMC9129054; doi:10.1371/journal.pone.0266856)
Supplement: S1 Appendix — (PDF) [file pone.0266856.s001.pdf]

S1 Appendix. Explanation of online survey questionnaires

Section 1. Demographic information

1. Gender (Male / Female)

2. Birth year (yyyy)

3. What is your major? (   )

4. Are you willing to participate in future visitor experiments? (Yes / No)

Section 2. Level of curiosity for visitor-based social contextual information

(5-point Likert scale; 1: *not at all* to 5: *very much*)

1. Comments on artwork   (   )
    i. Reason: (   )
2. Emotional response of others   (   )
    i. Reason: (   )
3. Artwork features from others   (   )
    i. Reason: (   )
4. Interest in artwork   (   )
    i. Reason: (   )
5. Understanding of artwork   (   )
    i. Reason: (   )
6. Liking of artwork   (   )
    i. Reason: (   )
7. Viewing time of artwork   (   )
    i. Reason: (   )
8. Proportion of visitors who viewed a specific artwork   (   )

i. Reason: ( )

9. Revisit count for the artwork ( )
   i. Reason: ( )
10. Most popular artworks ( )
    i. Reason: ( )
11. Exhibition comments ( )
    i. Reason: ( )
12. Visitor type compared with others ( )
    i. Reason: ( )
13. Exhibition satisfaction ( )
    i. Reason: ( )
14. Less popular artworks ( )
    i. Reason: ( )
15. Average viewing time for artworks ( )
    i. Reason: ( )
16. Average proportion of visitors who viewed the artworks ( )
    i. Reason: ( )
17. Average revisit counts for the artworks ( )
    i. Reason: ( )

Section 3. Art interest

We used the English version of VAIAK [1] to measure the art interest level of participants. Each question was assigned 7 points, with a maximum score of 77 points.

(7-point Likert scale; 1: *not at all* to 7: *very much*)

1. I enjoyed visiting art class in school

2. I enjoy talking about art with others
3. I have many friends/acquaintances who are interested in art
4. I am interested in art
5. I am always looking for new artistic impressions and experiences
6. During my everyday life I spontaneously notice art objects that I find fascinating
7. I come from a family that is interested in art

(7-point Likert scale; 1: *less than once per year* to 7: *once per week or more often*)

8. How often do you visit art museums or art galleries on average?
9. How often do you read books, magazines or catalogues about art?
10. How often do you view images of artworks (picture books, internet, etc.)?
11. How often do you visit events about art or art history (seminars, projects, festivals, etc.)?

Section 4. Art knowledge

Because we organized the experimental exhibition to select Korean contemporary artists (Ungno Lee), the questionnaire contained items measuring participant's knowledge about Korean contemporary art, similar to Belke, Leder, and Augustin [2]. Based on the consulting of a curator who has a lot of experience in art museum exhibition planning, we selected 10 representative artists related to Korean contemporary art and prepared the explanation of them. The survey participants were asked to choose an author who corresponds to a given explanatory text, and each question was assigned 10 points (maximum score = 100).

The list of selected Korean contemporary artist is like below:

1. Lee Jung-seob (1916 ~ 1956)
2. Lee Ung-no (1904 ~ 1989)
3. Kim Whanki (1913 ~ 1974)
4. Park Seo-bo (1931 ~)
5. Lee U-Fan (1936 ~)
6. Paik Nam June (1932 ~ 2006)
7. O Yoon (1946 ~ 1986)
8. Lee Bul (1964 ~)
9. Yang Haegue (1971~)
10. Suh Do-ho (1962~)

References

1. Specker E, Forster M, Brinkmann H, Boddy J, Pelowski M, Rosenberg R, et al. The Vienna Art Interest and Art Knowledge Questionnaire (VAIAK): A unified and validated measure of art interest and art knowledge.
2. Belke B, Leder H, Augustin MD. Mastering style. Effects of explicit style-related information, art knowledge and affective state on appreciation of abstract paintings. Psychol Sci. 2006;48(2):115-134.
