# Supplementary material for: The influence of visitor-based social contextual information on visitors’ museum experience
Source: PLoS One. 2022 May 24;17(5):e0266856. doi: 10.1371/journal.pone.0266856 (PMC9129054; doi:10.1371/journal.pone.0266856)
Supplement: S2 Appendix — (PDF) [file pone.0266856.s002.pdf]

S2 Appendix. Explanation of post-experiment interview

Participants were asked to evaluate exhibition and app and to do interview in visitor experiment. Visitor evaluation was conducted "after viewing each exhibition in the free-viewing section (block 2)", and the other is post-experiment interview.

Table A. Questions of Visitor evaluation after viewing each exhibition

	No.	Element
Satisfaction of exhibition [1]	1	Overall, I am satisfied with my experience at this exhibition.
	2	My decision to visit this exhibition was a wise one.
	3	Overall, compared to other exhibitions, I am satisfied with this exhibition.
Satisfaction of mobile app [2]	1	I am very contented with the mobile guidance app.
	2	I am very pleased with the mobile guidance app.
	3	I am very delighted with the mobile guidance app.
	4	All things considered, I will definitely recommend this mobile guidance application to my friends.

(5-point Likert scale; 1-not at all to 5-very much)

Table B. Post-experiment interview

No.	Question
(Case 1)	*When participant entered in interview room, ask him/her about the exhibition experience with mobile apps.*
1	"Was there any difference in the exhibition experience between the two mobile guidance applications?"

2	"Which of the two mobile applications do you prefer? What is the reason?"
3	"Are there any features that you would like added or modified to this mobile guidance app?"
(Case 2)	*Show the mobile guidance application with VSCI.*
4 ~ 16	For each component of VSCI*<br>(1) "How much do you prefer this information element?" (5-point Likert scale)<br>(2) "Why do you prefer (or not) this information element?"
(Case 3)	*Before finishing the interview,*
17	"Do you have any additional opinions about this experiment?"
18	"Thank you for participating into this experiment. Lastly, do you have any questions about this experiment?"

** Components of VSCI = { 'Exhibition comments', 'Preference score of exhibition', 'Most popular artworks', 'Comments on artwork', 'Emotional response of others', 'Art features noticed by others', 'Liking of artwork', 'Interest in artwork', 'Understanding of artwork', 'Viewing time of artwork', 'Revisit count for the artwork', 'Proportion of visitors who viewed', 'Visitor type compared with others' }*

References

1. Han H, Hyun SS. Key factors maximizing art museum visitors' satisfaction, commitment, and post-purchase intentions. Asia Pacific J Tour Res. 2017. doi:10.1080/10941665.2017.1345771
2. Song J, Kim J, Jones DR, Baker J, Chin WW. Application discoverability and user satisfaction in mobile application stores: An environmental psychology perspective. Decis Support Syst. 2014. doi:10.1016/j.dss.2013.10.004
