# Supplementary material for: The influence of visitor-based social contextual information on visitors’ museum experience
Source: PLoS One. 2022 May 24;17(5):e0266856. doi: 10.1371/journal.pone.0266856 (PMC9129054; doi:10.1371/journal.pone.0266856)
Supplement: S3 Appendix — (PDF) [file pone.0266856.s003.pdf]

S3 Appendix. Explanation of materials for visitor experiment

Table A. List of Artworks in first exhibition [E1]

No.	Title (year)	Size	Material
1	The pulse of nature (1950)	w-68 x h-133 cm	Ink on Korean paper
2	Drunken night (1955)	w-55 x h-40 cm	Ink and color on Korean paper
3	Composition (1961a)	w-89 x h-116 cm	Korean paper collage on canvas
4	Composition (1962)	w-70 x h-133 cm	Oil on canvas
5	Composition (1970)	w-65 x h-120 cm	Cotton on Korean paper
6	Composition (1972)	w-132 x h-274 cm	Ink on Korean paper
7	People (1986)	w-266 x h-167 cm	Ink on Korean paper
8	People (1988)	w-98 x h-177 cm	Ink on Korean paper
9	Eastern travel (1980)	w-18 x h-18 cm	Ink on Korean paper
10	Eastern travel (1980)	w-17 x h-17.5 cm	Ink on Korean paper

** All artworks are created by Lee-Ungno*

Table B. List of Artworks in second exhibition [E2]

No.	Title (year)	Size	Material
1	Growth (1950)	w-68 x h-133 cm	Ink and color on Korean paper
2	Heeve, ho! Heeve, ho! (1955)	w-44 x h-24.5 cm	Ink and color on Korean paper
3	Composition (1961b)	w-89 x h-116 cm	Korean paper collage on canvas
4	Composition (1963)	w-128 x h-66 cm	Ink and color on Korean paper
5	Composition (1972)	w-316 x h-269 cm	Ink and color on Korean paper
6	64 characters in the Book of the changes (1974)	w-24 x h-33 cm	Ink on Korean paper

7	People (1988)	w-70 x h-130 cm	Ink on Korean paper
8	People (1987)	w-40.5 x h-34.5 cm	Ink on Korean paper
9	Eastern travel (1980)	w-34 x h-34 cm	Ink on Korean paper
10	Eastern travel (1980)	w-39 x h-28 cm	Ink on Korean paper

** All artworks are created by Lee-Ungno*

Figure C. Preparations for experiment

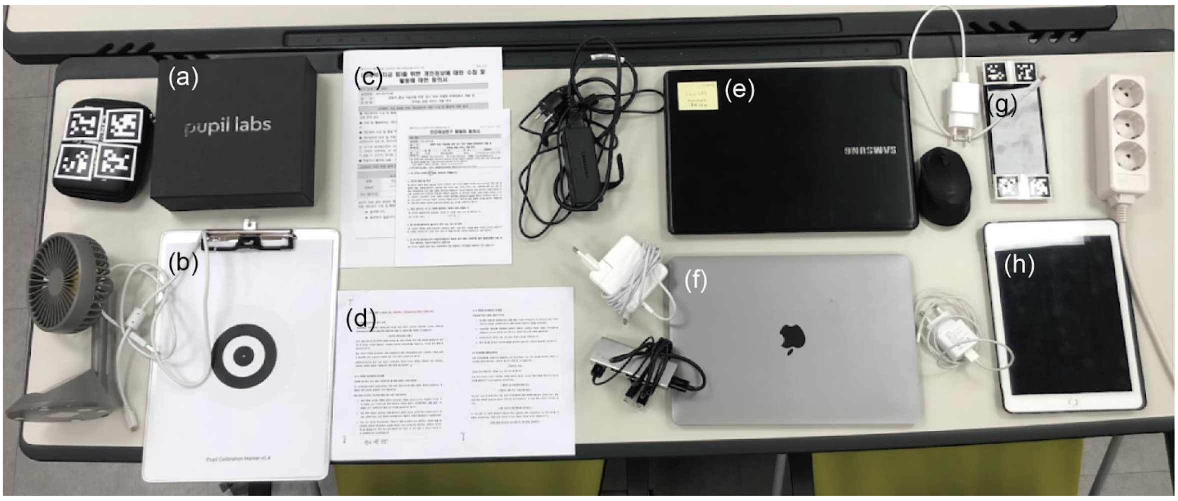

(a) Mobile eye-tracker (Pupil Labs, Pupil Core 120 Hz binocular)

(b) Calibration marker v.0.4. (Available from: https://docs.pupil-labs.com/pdfs/v0.4_marker.pdf)

(c) Consent form (KAIST IRB)

(d) Check-list of experiment for researchers

(e) First laptop computer (Samsung, NT series, OS: Windows 10) using as server of mobile application

(f) Second laptop computer (Apple, MacBook Pro 2018, OS: Catalina, 10.15) connected with mobile eye-tracker with Pupil Capture v.3.4 for tracking participants' eye-movement. (Available from: https://docs.pupil-labs.com/core/)

(g) Mobile phone (Samsung Galaxy Note 9, OS: Android 10) to display guidance application

(h) Tablet computer to introduce experiment to participants
