# Supplementary material for: The influence of visitor-based social contextual information on visitors’ museum experience
Source: PLoS One. 2022 May 24;17(5):e0266856. doi: 10.1371/journal.pone.0266856 (PMC9129054; doi:10.1371/journal.pone.0266856)
Supplement: Attachment — Submitted filename: Response_to_Reviewers.docx [file pone.0266856.s005.docx]

Dear Editor and Reviewers,

We were pleased to have an opportunity to revise our manuscript titled “**The influence of visitor-based social contextual information on visitors’ museum experience**.” We thank you for reading our manuscript thoroughly and offering constructive feedback. We are very grateful for your comments and have tried our best to address them by commenting below and highlighting the changes made in the revised manuscript. Both our comments below and the changes in the main document are highlighted in purple. We hope we have addressed all the comments and that the manuscript is now suitable for publication in PLoS ONE.

Sincerely,

On behalf of all authors,

Ji-Hyun Lee

**Editor:**

We thank the editor for supporting our work and for the invitation to submit a revised version.

2. Please amend your current ethics statement to address the following concerns:

a) Did participants provide their written or verbal informed consent to participate in this study?

b) If consent was verbal, please explain i) why written consent was not obtained, ii) how you documented participant consent, and iii) whether the ethics committees/IRB approved this consent procedure.

We added an ethics statement as follows:

(1) The participants were provided with a written consent form, and we collected the completed forms. Therefore, we updated “written” in the Procedure section (Line 355)

**Reviewer #1:**

First, we are grateful for your detailed review. We tried to enhance the quality of our research paper based on your valuable comments.

[1] Abstract

1. Why is there a need to focus more on the interactions between personal and social contexts? The research justification was unclear.

Answer:

We attempted to clarify these statements as follows:

(a) We revised this sentence, “yet only a few have focused on the interactions” to

“yet the interactions between personal and social contexts have not been fully researched.”

(b) We also added the following to lines 15–17 in the Abstract:

“Since previous studies related to these interactions have focused on the face-to-face conversation of visitor groups, attempts to provide the social information contributed by visitors have not progressed.”

[2] Introduction

2. Social information has been widely studied. Why is there a need for this study? Please justify the research question/gap/significance/contribution.

Answer:

Currently, most art museums have not been tried to provide social information to visitors of both offline and online exhibitions. In addition, in terms of social contexts, previous studies have focused on face-to-face conversations between visitors and have been limited to group interactions. Therefore, this research investigates the possibilities of providing visitor-contributed social information to visitors and examines the influence of the information on visiting experience. We rewrote some parts of the Introduction and added two references, as follows (Lines 46–60):

“However, the interactions between personal and social contexts are not fully researched. Some related studies have focused on communication between accompanying visitors in terms of social contexts [7,8]. These studies have concentrated on face-to-face conversations between visitors, and there has been insufficient research investigating social interaction from the various visitors to art museums. When forming an opinion in daily life, people are affected by the opinions or reactions of others, which can cause them to change their opinions. Such social information is essential data in almost all product, content, or service areas [9–11]. Through this social process that considers the experiences of ourselves and others together, we can identify the strengths and weaknesses of specific items, further create meaning, and make decisions [12,13]. However, there have been few attempts to provide supplemental information on visitors’ thoughts or reactions to exhibitions in museums and examine the effectiveness of utilizing such information. Regarding supplemental information, the existing research mainly focuses on content written by experts [14,15]. Thus, to fill the gap in the literature, this study aimed to investigate the influence of the social information contributed by individuals in art museums.”

*- Added references:*

[12] Basu C, Hirsh H, Cohen W. Recommendation as classification: using social and content-based information in recommendation. In: Proc 15th Nat Conf on Artificial Intelligence. Madison (1998. pp. 714–720).

[13] Godes D, Silva JC. Sequential and temporal dynamics of online opinion. Marketing Science. 2012. doi:10.1287/mksc.1110.0653

3. The research contributions were unclear. What does the study provide with respect to others?

Answer:

A main contribution of our research is that it reveals the possibility of enhancing the visitor experience via social information, VSCI. Therefore, we tried to update the last part of the Introduction (Lines 61–69) by adding:

“Looking at museums as a service product [16], social information can help to provide a better visitor experience in a social context. To do so, we identified the categories and components of social information via a literature review. Based on the identified social information, we investigated the visitors’ needs and opinions to design the museum guidance application and developed an app to deliver the social information. Additionally, we experimented with the influence of visitor-contributed social information using a mobile eye-tracker. In summary, this study attempted to reveal the possibility of enhancing the visitor experience via social information. Further, we proposed a method of providing social information to enrich the visitor experience through museum guidance applications.”

[3] Literature Review

4. On page 4 lines 67-68, “However, research using various data collected to benefit visitors (rather than museum experts) is insufficient.”, what did you mean about this?

Answer:

Based on the advancement of technologies, contemporary art museums and researchers have collected visitors’ behavioral and emotional data to understand visitors. However, these data have not been provided to visitors. Our starting point of this research was the question, “Why don’t museums provide visitors with the numerous data collected from visitors?” Subsequently, we revised this as follows (Lines 78–81):

“Even though visitor behavior and reaction data have been collected, these collected data have been used as information sources for researchers or museum operators only and have not been provided to museum visitors.”

5. The examples of social information on pages 4-5 were unclear.

6. Please define each variable/concept of the study (conceptualization and operationalization) in the literature review.

7. Research questions/hypotheses were not well presented in the literature review.

8. It was unclear how you determined and proposed the elements of VSCI. A conceptual framework discussion is recommended.

Answer:

Based on these comments, we realized that our explanation of VSCI has some problems. As you suggested, we added a conceptual framework of VSCI with further explanations. Our revisions are as follows:

(a) We updated the first explanation of VSCI in the literature review and provided two research questions (Lines 98–106):

“To overcome these limitations, we proposed using “visitor-based social contextual information” (VSCI), the social information individuals provide—feedback, reactions, or behavioral data—can be applied to facilitate interactions in a social context. VSCI is a collation of visitors’ behaviors and reaction data in museums and provides visitors with information to enhance their museum experience. Therefore, our research questions are as follows:

- RQ 1. What are the components of social information from other visitors in art museums?
- RQ 2. Are visitors’ museum experiences enhanced when social information from others is provided to visitors?”

(b) We changed the section title, “Components of visitor-based social contextual information” to “Conceptualization of visitor-based social contextual information.” (Line 108)

(c) A conceptual framework was added with detailed information (Lines 109–122), and A new figure (Fig.1.) was added:

“To answer the first research question, we identified the types of data collected from previous visitor studies to determine the information elements constituting the VSCI. We reviewed the previous visitor studies from interdisciplinary research to investigate visitors’ behaviors, experiences, reactions, and thoughts systematically. From the literature review, we identified five categories with detailed components. First, visitor evaluation was divided into level of satisfaction with exhibitions and museum objects. Second, visitor behavioral data were collected, such as museum object viewing time, visiting time, and visitor type based on the behavioral characteristics. Third, many studies have paid attention to the visitors’ emotions during the art appreciation process. Fourth, some studies focused on museum objects’ characteristics to which the visitors reacted sensitively. Lastly, the effect of visitors’ comments on their museum experience were analyzed. Subsequently, we established the VSCI with the commonly discussed factors from the literature review, as illustrated in Fig 1.

**Fig. 1.** Conceptual framework of VSCI based on previous visitor studies.”

[4] Methodology

9. I suggest the author(s) have a section to discuss the research design. Is there any reference to support your research design and process?

Answer:

We designed the research process with reference to the prototype-based studies: (1) insight research (or user requirements); (2) app or system design and prototyping; and (3) testing or experiment. As you suggested, we updated the explanation with two additional references [65,66] in Lines 171–182:

“the research procedure was designed with reference to prototype-based studies that have designed and developed apps or systems by analyzing user requirements [65,66]. Additionally, an experiment was planned to confirm the differences in visitor experiences depending on whether VSCI was provided or not. Therefore, we designed a research process with three stages: (1) insight research to collect visitors’ VSCI needs and opinions; (2) museum application design and prototyping based on the derived insights; and (3) visitor experiments to reveal the impact of VSCI on the visitor experience.”

- Added references:

[65] Kang B, Song B, Yang S, Lee J. A Development Technique for Mobile Applications Program. Applied Computing and Information Technology 2017 (pp. 47–62). Springer, Cham. doi:[10.1007/978-3-319-51472-7_4](https://doi.org/10.1007/978-3-319-51472-7_4)

[66] Liedtke C, Baedeker C, Hasselkuß M, Rohn H, Grinewitschus V. User-integrated innovation in Sustainable LivingLabs: An experimental infrastructure for researching and developing sustainable product service systems. J Clean Prod. 2015. doi:[10.1016/j.jclepro.2014.04.070](https://doi.org/10.1016/j.jclepro.2014.04.070)

10. In stage 1, the sample size was small. How did you determine the proper sample size? What were the qualifications of respondents? Were they domestic visitors? How and when did you collect data? Why did you use an online survey since it has several limitations?

Answer:

(a) Based on this valuable comment, we found that we should explain this limitation carefully to the reviewer(s) and reader(s). In this study, an online survey was performed to understand the design insights and user demands about social information for mobile museum guidance apps (e.g., *Gugenheimer et al.** performed an online survey (n = 48) for gaining user demand). This process corresponds to the initial stage of the method for developing a mobile museum app considering VSCI, and our method can be used by future researchers interested in VSCI. However, the survey results in Stage 1 are not constant, and the probability of the differences could be because of visitors’ characteristics, such as ages, visit groups, or countries. Therefore, we added this limitation in the “Limitations and future research” section with two additional references [96,97], as follows (Lines 650–654):

“Second, even though the purpose of the online survey in Stage 1 was finding insights to design a mobile guidance application with VSCI, the sample size was quite small (n = 71). In addition to the purpose of understanding user demand, in-depth research is required in consideration of various visitor characteristics (e.g., age differences [39], the composition of the visit groups [95], and countries [96]) to derive standardized VSCI.”

** Gugenheimer J, Stemasov E, Frommel J, Rukzio E. Sharevr: Enabling co-located experiences for virtual reality between hmd and non-hmd users. Proceedings of the 2017 CHI Conference on Human Factors in Computing Systems (CHI ‘17). ACM, New York, NY, USA, 4021-4033. doi:* [*10.1145/3025453.3025683*](https://doi.org/10.1145/3025453.3025683)

*- Added references:*

[96] Sandifer C. Time‐based behaviors at an interactive science museum: Exploring the differences between weekday/weekend and family/nonfamily visitors. Science Education. 1997. doi:10.1002/(SICI)1098-237X(199711)81:6<689::AID-SCE6>3.0.CO;2-E

[97] Rawlings D, Vidal N, Furnham A. Personality and aesthetic preference in Spain and England: Two studies relating sensation seeking and openness to experience to liking for paintings and music. European journal of personality. 2000. doi:[10.1002/1099-0984(200011/12)14:6<553::AID-PER384>3.0.CO;2-H](https://doi.org/10.1002/1099-0984(200011/12)14:6%3c553::aid-per384%3e3.0.co;2-h)

(b) We updated the explanation of Stage 1 in the Methods section, as follows (Lines 203–205):

“An online survey was chosen to obtain opinions from the various visitors who had experience visiting art museums and using mobile guidance services.”

We also added the following clarification in Lines 205–206:

“The survey responses were collected via Google Forms from May 24 to June 8, 2021.”

And in Line 216:

“from multiple online communities in South Korea (e.g., ARA and Hongik-in).”

11. In stage 3, how did you determine the proper sample size? What were the qualifications of participants? How did you recruit them? When did you conduct the experiment?

Answer:

(a) The quantitative statistics studies need to “test at least 20 users to get statistically significant numbers (*Nielson Norman group, 2012**).” Also, referring to the sample size of these previous studies (Brieber et al., 2014 [95]; Paasschen et al., 2015 [49]) that conducted eye tracking experiments on museum visitors, we determined the number of samples (n = 20) for each condition.

** Nielsen Norman Group (2012). How Many Test Users in a Usability Study? retrieved from https://www.nngroup.com/articles/how-many-test-users/*

(b) We revised and updated some parts of Stage 3, as follows (Lines 329–330):

“We contacted those who agreed to participate in this experiment via the online survey.”

(Error found) “male:female = 19:21” in Line 331.

“This experiment was conducted from August 6 to August 21, 2021, as three blocks (Fig 4) and took about 90 mins, on average.” (Lines 353–354)

12. Where was the discussion of stage 4 in the methodology? Were interviews in stage 4? Who were the interviewees?

Answer:

We found the statement problems in describing the post-experiment interview. The interviews were conducted in the third block of Stage 3 (Fig. 4; see 368–378 lines); however, our figures did not describe it. Therefore, we changed the two Figures (Fig. 2 and Fig. 4) and some parts of the manuscript as below:

(a) “four stages” to “three stages” in Line 179.

(b) Changing Figure 2 and 4 to mention the post-experiment interview in Line 198 (Fig. 2) and Line 377 (Fig. 4);

(c) Adding “with a post-experiment interview” in Line 190.

[Results]

13. Often, visitor satisfaction and behavior are affected by museum interpreters or tour guides. Have you considered the impact of the interactions between them? Were they individual/independent visitors or visitors from a package tour?

Answer:

(a) When VSCI was delivered to individual visitors through the mobile museum guidance application, we paid attention to the impact on their museum experience. (e.g., Line 621: “This study focused mainly on mobile guidance applications”). Therefore, the docent program was not considered in this study.

(b) In the visitor experiment, all participants were independent visitors. As you commented, the visiting groups can have different experiences compared to the VSCI. For this reason, we updated this information in the Limitations and Future research sections, as follows (Lines 652–654):

“in-depth research is required in consideration of various visitor characteristics (e.g., age differences [39], **the composition of the visit groups** [95], and countries [96]) to derive standardized VSCI.”

[Discussion and Implications]

14. Some findings were consistent with previous studies. What is the difference between the current paper and prior works? Please strengthen the uniqueness of the study.

Answer:

We understand this concern. We found two parts that do not mention the differences compared to previous works. We revised these two parts as follows (Lines 486–491 and Lines 561–565):

(a) “This result is similar to Temme [87], who indicated that although audiences want more information related to artwork, providing more information does not increase their viewing time. In addition, Temme [87] demonstrated that as the amount of information increases, the enjoyment of artworks decreases, and there is no difference in interest in art labels. On the contrary, when VSCI was presented, both the satisfaction of the exhibition and mobile app increased.”

(b) “This relates to Screven’s [88] results, who reported that it is necessary to provide information to address the problem of visitors struggling to determine why the artworks exhibited in art museums are important to them. In other words, it implies that VSCI, which represents the others’ art experience, can play a role in improving the understanding of artworks.”

15. This study failed to answer the “so what” question. Please strengthen the theoretical and practical implications of this study.

Answer:

As you suggested, we strengthened the theoretical and practical implications or contributions in three points:

(a) For academic interest (Lines 672–674):

“The contributions of this research are twofold. For academic interest, there is the need for an in-depth understanding of visitor-oriented social information in the field of visitor studies.”

(b) For practical interest (Lines 685–687):

“For practical interest, we proposed a method of designing and developing a mobile museum guidance application, including VSCI. This method could help museums use social information as mobile app content.”

(c) Additional summary (Lines 691–693):

“In summary, this study suggested one possible approach of providing social information for the visitor-centered design of museum guidance applications, which contributes to the field of visitor studies.”

**Changes to the reference list:**

Based on reviewers comments, we cited six additional articles:

[12] Basu C, Hirsh H, Cohen W. Recommendation as classification: using social and content-based information in recommendation. In: Proc 15th Nat Conf on Artificial Intelligence. Madison (1998. pp. 714–720).

[13] Godes D, Silva JC. Sequential and temporal dynamics of online opinion. Marketing Science. 2012. doi:10.1287/mksc.1110.0653

[65] Kang B, Song B, Yang S, Lee J. A Development Technique for Mobile Applications Program. Applied Computing and Information Technology 2017 (pp. 47–62). Springer, Cham. doi:[10.1007/978-3-319-51472-7_4](https://doi.org/10.1007/978-3-319-51472-7_4)

[66] Liedtke C, Baedeker C, Hasselkuß M, Rohn H, Grinewitschus V. User-integrated innovation in Sustainable LivingLabs: An experimental infrastructure for researching and developing sustainable product service systems. J Clean Prod. 2015. doi:[10.1016/j.jclepro.2014.04.070](https://doi.org/10.1016/j.jclepro.2014.04.070)

[96] Sandifer C. Time‐based behaviors at an interactive science museum: Exploring the differences between weekday/weekend and family/nonfamily visitors. Science Education. 1997. doi:10.1002/(SICI)1098-237X(199711)81:6<689::AID-SCE6>3.0.CO;2-E

[97] Rawlings D, Vidal N, Furnham A. Personality and aesthetic preference in Spain and England: Two studies relating sensation seeking and openness to experience to liking for paintings and music. European journal of personality. 2000. doi:[10.1002/1099-0984(200011/12)14:6<553::AID-PER384>3.0.CO;2-H](https://doi.org/10.1002/1099-0984(200011/12)14:6%3c553::aid-per384%3e3.0.co;2-h)
